# TAF6δ Controls Apoptosis and Gene Expression in the Absence of p53

**DOI:** 10.1371/journal.pone.0002721

**Published:** 2008-07-16

**Authors:** Emmanuelle Wilhelm, François-Xavier Pellay, Arndt Benecke, Brendan Bell

**Affiliations:** 1 RNA Group, Département de microbiologie et d'infectiologie, Faculté de médecine et sciences de la santé, Université de Sherbrooke, Sherbrooke, Québec, Canada; 2 Institut des Hautes Études Scientifiques and Institut de Recherche Interdisciplinaire – CNRS USR3078 - Université de Lille, Bures sur Yvette, France; University of Munich and Center of Integrated Protein Science, Germany

## Abstract

**Background:**

Life and death decisions of metazoan cells hinge on the balance between the expression of pro- versus anti-apoptotic gene products. The general RNA polymerase II transcription factor, TFIID, plays a central role in the regulation of gene expression through its core promoter recognition and co-activator functions. The core TFIID subunit TAF6 acts *in vitro* as an essential co-activator of transcription for the p53 tumor suppressor protein. We previously identified a splice variant of TAF6, termed TAF6δ that can be induced during apoptosis.

**Methodology/Principal Findings:**

To elucidate the impact of TAF6δ on cell death and gene expression, we have employed modified antisense oligonucleotides to enforce expression of endogenous TAF6δ. The induction of endogenous TAF6δ triggered apoptosis in tumor cell lines, including cells devoid of p53. Microarray experiments revealed that TAF6δ activates gene expression independently of cellular p53 status.

**Conclusions:**

Our data define TAF6δ as a pivotal node in a signaling pathway that controls gene expression programs and apoptosis in the absence of p53.

## Introduction

Tightly controlled programmed cell death is essential for the development and tissue homeostasis of all animals. Metazoan cells possess an evolutionary conserved network of proteins (e.g. caspases, Bcl-2 family members and death receptors) that execute appropriate life or death decisions in response to extrinsic and intrinsic cellular cues [1]. Deregulated apoptosis underlies numerous human disease states including neurodegenerative disorders and cancer [2]. The identification and molecular characterization of the critical control points in apoptotic pathways is therefore essential to reveal novel therapeutic avenues to treat a broad array of human pathologies.

Gene expression plays a key role in the response of cells to death-inducing stimuli. A growing body of evidence indicates that the levels of numerous death-related genes can be induced during apoptosis [3]. The integration of cellular signals from diverse apoptotic pathways requires the finely balanced expression of pro- versus anti-apoptotic proteins. Gene expression patterns of pro- and anti-apoptotic genes, established by the levels of transcription [4] as well as alternative splicing [5], can dictate the life-or-death decisions of cells. The most intensely studied protein known to control apoptosis by altering gene expression is the p53 tumor suppressor. Current paradigms link the p53 tumor suppression function to its capacity to induce apoptosis in response to genotoxic stress [6]. The pro-apoptotic activity of p53 depends largely on its function as a transcriptional activator that binds directly to the promoters of pro-apoptotic genes including *FDXR* [7], [8], *PUMA* [9], *Noxa* [10], Bax [11] and *p53AIP1* [12].

TFIID is a multi-protein complex that plays a pivotal role in the transcription of protein-coding genes in eukaryotes. TFIID is composed of the TATA-binding protein (TBP) and up to 14 evolutionarily conserved TBP-associated factors (TAFs) [13], [14]. TFIID can play a rate-limiting role in the regulation of transcription through the recognition of the core promoter elements such as the TATA-box, the initiator, and downstream promoter element (DPE) [15]. The TFIID complex also engages in direct contacts with DNA-binding transcriptional factors to co-activate gene expression [16]. The architecture and integrity of TFIID complexes depends on a network of TAF-TAF interactions that are predominantly mediated by dimerization of TAFs via their interlocking histone fold motifs [17]. Histone-fold pairs within TFIID include TAF6-TAF9 [18], TAF4-TAF12 [19], TAF11-13 [20], TAF8-TAF10 [21] and TAF3-TAF10 [22]. TAF4 and TAF5 together with the histone fold containing TAF12, TAF9 and TAF6 are defined as core TAFs, since their depletion from *Drosophila* cells results in overall destabilization of TFIID complexes [23].

The core TFIID subunit TAF6 (hTAF_II_70/80) has been shown to be broadly required for RNA polymerase II (Pol II) transcription in yeast when total poly(A)^+^ mRNA levels were monitored [24]. A more recent microarray analysis estimated that approximately 18% of the yeast Pol II transcriptome depends on TAF6 [25]. TAF6 has been shown to be essential for viability in yeast [24], [26], plants [27], insects [28] and fish [29]. The requirement for TAF6 in all model organisms studied, together with the fact that human cells have a single *TAF6* gene [30], [31], strongly implies that TAF6 is essential for human cell viability.

TAF6 and TAF6-TAF9 dimers can bind to the downstream promoter element (DPE) [32], [33]. In addition to its core promoter recognition function, TAF6 can also interact with transcriptional activators. For example, *in vitro* experiments have shown that both TAF6 [34], and its dimerization partner TAF9 [35], interact directly with p53 and are required for the activation of transcription by p53. The available evidence implies that the TAF6-p53 interaction is required for the activation of at least some, and potentially all, p53 target genes *in vivo* [36], [37], [38].

We have identified and characterized a splice variant termed TAF6δ that lacks 10 amino acids in the centre of its histone fold domain [39]. TAF6δ is unable to interact with TAF9, but retains interactions with other TFIID subunits. TAF6δ expression is induced in promyelocytic HL-60 cells undergoing retinoic-acid dependent apoptosis. TAF6δ overexpression induces apoptosis in HeLa cells, evoking the possibility that TFIID function could be coupled to certain apoptotic pathways via TAF6δ. Importantly, however, it is not currently known if changes in the expression of endogenous TAF6δ can influence tumor cell death. Furthermore, despite the physical and functional interactions between the pivotal tumor suppressor p53 and TAF6 [34], nothing is currently known about whether p53 is required for TAF6δ-mediated apoptosis. Here, we have used splice-site switching modified antisense RNA technology to demonstrate that endogenous TAF6δ controls apoptosis and that p53 is not required for TAF6δ-dependent apoptosis or TAF6δ-dependent gene expression.

## Materials and Methods

### Cell culture

HeLa cells were grown in DMEM containing 2.5% CS and 2.5% FCS. Saos-2 and H1299 cell lines were cultured in DMEM with 10% FCS. A549 cells were grown in Ham's F12 medium with 10% FCS. HCT-116 cells were grown in McCoy's media supplemented with 10% FCS. When indicated, cells were treated with the proteasome inhibitor MG-132 (Calbiochem) at 0.5 µM and/or pan-caspase inhibitor Z-VAD-FMK (Biomol) at 100 µM.

### Transfections

Oligonucleotides were transfected with lipofectamine 2000 (Invitrogen) as a delivery agent (1.6 µl/ml) according to the manufacturer's recommendations. 2′-O-methyl-oligoribonucleoside phosphorothioate antisense 20-mers were from Sigma-Proligo. “TAF6 AS1” 5′-CGAUCUCUUUGAUGCGGUAG-3′ targets the central 20 nucleotides of the alternative exon 2 of TAF6, “TAF6 AS2” 5′-GCCGGGUCACCUGUGCGAUC-3′ the constitutive alpha 5′ splice site. “Control AS” 5′-AUGGCCUCGACGUGCGCGCU-3′ is a scrambled oligo used as a negative control. “Bcl-x AS” 5′-ACCCAGCCGCCGUUCUCC-3′ targets the 5′-splice site of Bcl-x_L_ [40]. Plasmids were transfected using 1 µl DMRIE-C (Invitrogen) as a delivery agent in a 24 well plate according to the manufacturer's recommendations. All transfections were performed in OptiMEM medium (Invitrogen).

### RT-PCR

Total RNA was isolated from cells using Trizol (Invitrogen) according to the manufacturer's recommendations. 1 µg of total RNA was reverse transcribed using AMV-RT (Roche). 1/10 of the total cDNA was used per PCR reaction : 95°C, 3 min; 25 cycles of 94°C for 1 min, 58°C for 45 sec, 68°C for 50 sec; final extension at 68°C for 5 min with the following oligonucleotide pairs. For Taf6; forward 5′-ATGGGCATCGCCCAGATTCAGG-3′ and reverse 5′-AAGGCGTAGTCAATGTCACTGG-3′. For Bcl-x; forward 5′-TCATTTCCGACTGAAGAGTGA-3′ and reverse 5′-ATGGCAGCAGTAAAGCAAGCG-3′

### Apoptosis assays

Detection of caspase cleaved cytokeratin-18 by flow cytometry was performed using Cytodeath reagent (Roche) according to the manufacturer's recommendations. Flow cytometric analysis of sub-G1 DNA content was performed as described [39].

### Plasmids

To construct pASTAF6, the genomic region of *TAF6* containing exon2 to exon4 was amplified by PCR from HEK 293 genomic DNA with primers 5′-GGAGAAGAGGGACTCCAGAATGGCTG-3′ (forward) and 5′-TCCCCCAACCTTTGAGGCAGACG-3′ (reverse). The resulting product was digested with HindIII and SmaI and inserted into the same sites of the plasmid pXJ42hTAF_II_80α [39].

### Antibodies

Monoclonal antibodies directed against TAF6δ (37TA-1 & 37TA-2), TAF6α (25TA) [39], TBP (3G3) [41], and TAF5 (2D2) [42] have been described. Monoclonal antibodies against TAF6 and PARP-1 were purchased from BD Transduction Laboratories and Biomol, respectively.

### Immunocytochemistry

Cells were fixed in 4% PFA, permeabilized with PBS-0.1% Triton X-100 (PBS-Tx) and incubated for 30 min in blocking buffer (PBS-Tx containing 1% bovine serum albumin (BSA) and 0.5% fish gelatine (Sigma-Aldrich)). Cells were then sequentially incubated one hour at room temperature, followed by washes, with each of the following antibodies diluted in blocking buffer; anti-TAF6 mAb (1/400), Oregon Green goat anti-mouse IgG secondary antibody (Molecular Probes), anti-TAF6δ mAb (37TA-1: 1/1000), Alexa Fluor 546 goat anti-mouse IgG_1_ secondary antibody (Molecular Probes). Cells were then treated with Hoechst 33342 (2 µg/ml) and visualized by fluorescence microscopy.

### Microarray Analysis of Gene Expression

#### Transcriptome Acquisition

Total RNA was analyzed using ABI Human Whole Genome Survey Arrays v1.0 arrays (Prod. No.: 4359030), containing 31,700 60-mer oligonucleotide probes representing a set of 27,868 individual annotated human genes. Chemiluminescence detection technology is used to detect as little as a femtomole of expressed mRNA. One single round of linear amplification was performed from total RNA according to the Applied Biosystems RT-IVT (Applied Biosystems, ProdNo: 4339628) protocol using 2 µg of total RNA. cDNA synthesis, *in vitro* transcription and labeling, fragmentation, hybridization, staining, and scanning were performed as directed by the supplier (Applied Biosystems, ProdNo: 4346875).

#### Transcriptome Data Analysis

Applied Biosystems Expression Array System Software v1.1.1. (ProdNo: 4364137) has been used to acquire the chemiluminescence and fluorescence images and primary data analysis. We renormalized the resulting data according to the logarithmic signal median once more after having removed control probes and those probes for which the Applied Biosystems Software has set flags equal to or greater than 2^12^, indicating compromised measurements (as recommended by Applied Biosystems). Log_2_ subtractions were determined using averages over the weighted individual signal values. The weights are anti-proportional to the corresponding coefficient of variation. For these inter-assay comparisons the NeONORM method was used for normalization using sensitivity parameter *k* = 0.20 [43]. P-values were determined using a standard ANOVA method. Multiple probes for a single gene, cross-reactivity of a single probe to several genes, as well as the resolution of probe-ID annotations were done according to the standards defined previously [44]. Gene lists corresponding to statistically significant changes in expression (P<0.05) are available as supplementary data files: genes changing in response TAF6δ in HCT-116 p53 +/+ cells (Supplementary Data File S1), genes changing in response to TAF6δ in HCT-116 p53 −/− (Supplementary Data File S2), genes differentially expressed in the HCT-116 p53 +/+ cells versus HCT-116 p53 −/− cells both in cells treated with control oligonucleotide and TAF6δ-inducing oligonucleotides (Supplementary Data File S3), and genes differentially regulated by TAF6δ in both HCT-116 p53 +/+ cells and HCT-116 p53 −/− cells (Supplementary Data File S4).

The microarray data for the experiments described here were deposited in the Gene Expression Omnibus database (http://www.ncbi.nlm.nih.gov/geo/) under accession number: GSE10795.

The supplementary data files can also be accessed from the Benecke group webpage: http://seg.ihes.fr/.

### Real time PCR

Real Time PCR was performed on cDNA prepared as for microarray experiments (above) using ABI TaqMan® assays. The genes and their respective assay numbers were ATF3 (Hs 00231069_m1), ACRC (Hs 00369516_m1), FNBP4 (Hs 00392543_m1) HES1 (Hs 00172878_m1), and HOM-TES-103 (Hs 00209961_m1). Real-time PCR was performed on 10 ng of cDNA with 1.25 µl of 20× TaqMan® probes and 12.5 µl 2× TaqMan® Universal Master Mix (ABI) in a final 25 µl reaction. Real-time PCR relative quantification assay was running for 2 min at 50°C, 10 min at 95°C, followed by 40 cycles of 15 sec at 95°C and 1 min at 60°C on an ABI 7500 system. Relative quantity of target genes was calculated using the comparative C_T_ (ΔΔC_T_) method using FNBP4 as the internal control.

## Results

### Selective induction of endogenous TAF6δ mRNA expression by splice-switching oligonucleotides

**Figure 1:**
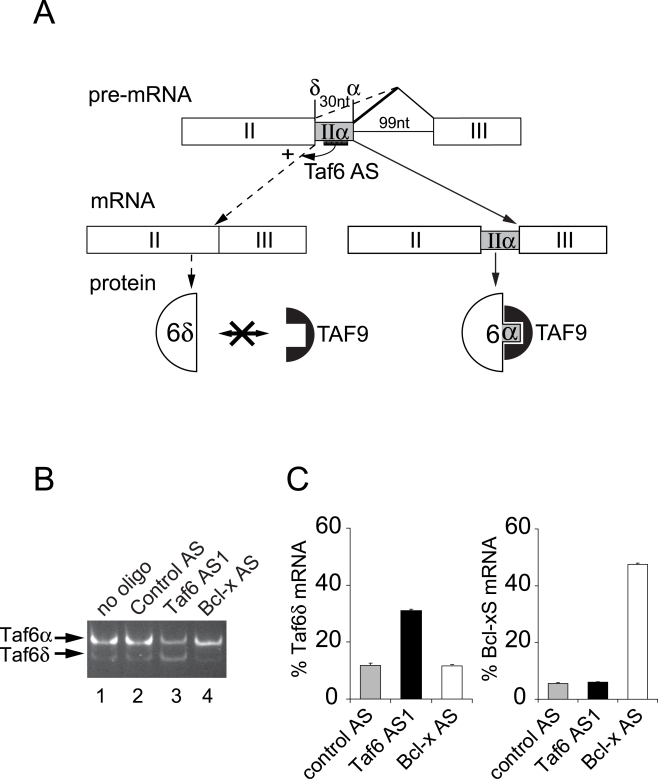
Specific control of endogenous TAF6 alternative splicing by modified antisense RNA oligonucleotides in living cells. (A) The region of the TAF6 pre-mRNA that includes two alternative 5′ splice sites (SSs) that produce either the constitutive α splice variant or the alternative δ splice variant is schematically depicted. Selection of an intron-proximal α 5′ splice site (SS) results in production of the α isoform of TAF6 (at right) whereas the selection of the proximal δ 5′ SS results in the production of the δ isoform (at left). The SSOs that base pair with the alternative exon forces splicing from the distal 5′ SS and induces expression of the endogenous TAF6δ isoform (at left). The protein produced by the major splice variant, TAF6α, can interact with the TFIID subunit, TAF9 via its histone fold domain. In contrast, TAF6δ lacks 10 amino acids of helix 2 of its histone fold motif and therefore cannot interact with TAF9. (B) Antisense RNA oligonucleotides induce endogenous TAF6δ mRNA expression. HeLa cells were transfected with 200 nM oligonucleotides directed against: the alternative exon II (exon IIα) of the TAF6 gene (Taf6 AS1), the Bcl-x gene (Bcl-x AS), or a scrambled control oligonucleotide (Control AS). 24 hours post-transfection total RNA was isolated and subjected to RT-PCR with primers that amplify both the TAF6α and the alternative TAF6δ mRNAs. (C) Specificity of TAF6 splice site switching oligonucleotides. HeLa cells were transfected with antisense RNA oligonucleotides as in A. RT-PCR was perfomed with primers sets that amplify the both the α and δ TAF6 splice variants, or both the Bcl-xS and Bcl-xL splice variants. PCR products were separated by microfluidity and analyzed using a 2100 Agilent bioanalyzer. The ratio of TAF6δ mRNA over total TAF6 mRNA and the ratio of Bcl-Xs mRNA over total Bcl-X mRNA are expressed on the y-axis. The values from cells treated with scrambled control (grey bars), Taf6 AS1 (black bars), or Bcl-X AS (white bars) are shown. Error bars represent the standard deviation of three independent transfections.

To date four splice variants of TAF6 have been identified and termed α, β, γ, and δ [31], [39], [45]. Here we focus on the TAF6δ splice variant due to its potentially important role in programmed cell death [39]. The total number of distinct TAF6 mRNA species produced by alternative splicing has not yet been established. For clarity, we therefore refer here collectively to all TAF6 splice variants lacking the 30 nucleotide exon IIα as TAF6δ and to TAF6α as all species of mRNA containing exon IIα (Fig. 1). The *TAF6* genomic locus shows that the major TAF6α isoform is produced by the selection of an intron proximal 5′ splice site (SS) (Fig. 1A). In contrast, the TAF6δ isoform is produced by an alternative splicing event at the intron distal 5′ SS (labelled δ in Fig. 1A).

To dissect the biological role of endogenous TAF6δ, we exploited splice-switching oligonucleotides (SSOs) [40], [46] to experimentally manipulate endogenous TAF6 alternative splicing. The HeLa cell system represents a natural cellular context to study TAF6δ function because the TAF6δ variant was originally cloned from a HeLa cell cDNA library [39]. We transfected HeLa cells with synthetic 2′-*O*-methyl-modified oligoribonucleoside phosphorothioate that hybridizes to the central 20 nucleotides of alternative exon IIα (Fig. 1A). The ratio of the alternative TAF6δ mRNA level with respect to TAF6α mRNA level was analyzed by RT-PCR of RNA samples from transfected HeLa cells. The transfection of the antisense oligonucleotide Taf6 AS1 resulted in a marked increase in the level of the TAF6δ mRNA and a concurrent decrease in the level of the major TAF6α mRNA (Fig. 1B, lane 3). In contrast, transfection of an oligonucleotide of scrambled sequence had no effect on the TAF6δ/TAF6α mRNA ratio (Fig. 1B, lane 2). To further demonstrate the specificity of the SSOs, we transfected antisense RNA oligonucleotides shown to enforce the expression of the Bcl-xS splice variant [40]. The TAF6δ/TAF6α+δ mRNA ratio was increased ∼3-fold by treatment with the Taf6 AS1 oligonucleotide, but unchanged by the Bcl-x AS oligonucleotide (Fig. 1C). Conversely the ratio of Bcl-xS/Bcl-xL+xS mRNA was increased ∼10-fold by Bcl-x AS but unaffected by Taf6 AS1 (Fig. 1C). Control RT-PCR reactions showed that ratio of TAF6δ with respect to total TAF6 mRNA is increased by Taf6 SSO but none of the ratios of any other known TAF6 alternative splice variants was affected (data not shown). The SSOs used therefore impact specifically on TAF6δ alternative splicing without influencing overall expression patterns of TAF6 mRNA. These results demonstrate that TAF6-directed SSOs are an efficient and selective method to enforce the expression of the endogenous TAF6δ mRNA in HeLa cells.

To further characterize the cellular response to TAF6 splice site switching antisense oligonucleotides we performed a time course analysis. The level of TAF6δ mRNA is detectably increased after 4 hours and increases until 24 hours (Supplementary Fig. S1A). These results are consistent with a previous study targeting the Bcl-x gene [40], and establish a kinetic framework to follow the early outcomes of TAF6δ mRNA expression in transfected cells. We next investigated the concentration dependence for the Taf6 response to treatment with the AS1 oligonucleotide. The induction of TAF6δ mRNA was observed with transfection of as little as 50 nM Taf6 AS1 and sharply increased until treatment with 200 nM Taf6 AS1, after which a plateau was reached (Supplementary Fig. S1B). Based on these results, we have employed 200 nM oligonucleotide concentrations herein, unless otherwise stated, for robust and specific induction of endogenous TAF6δ mRNA in living cells.

### Splice-switching oligonucleotides increase endogenous TAF6δ protein levels

**Figure 2:**
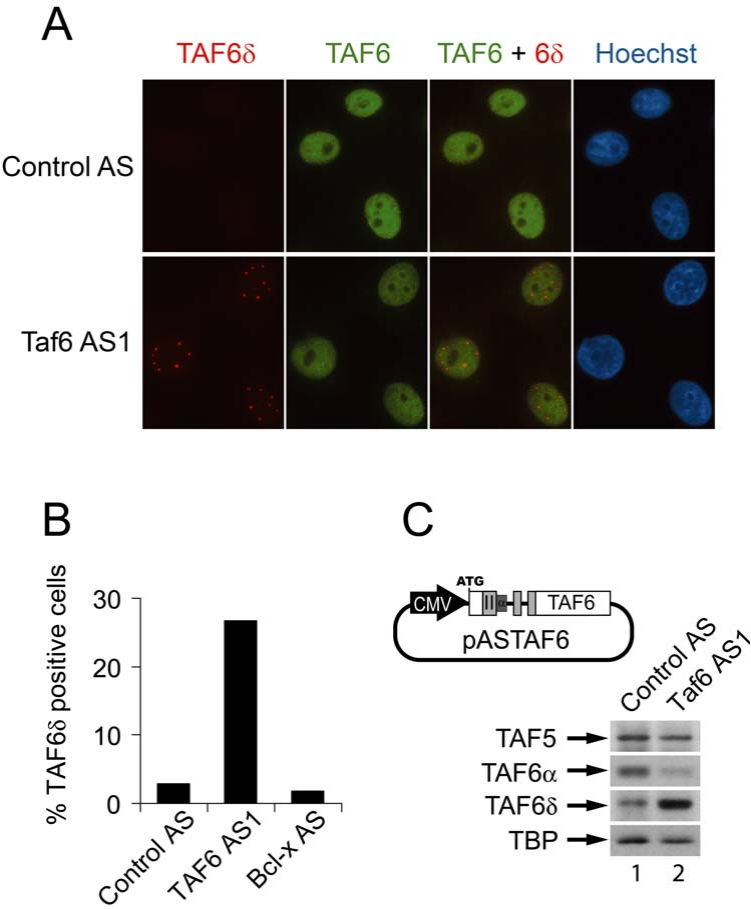
Specific induction of TAF6δ protein expression by modified antisense RNA oligonucleotides. (A) HeLa cells were transfected with splice site-switching oligonucleotides directed against exon IIα of the TAF6 gene and treated with MG-132 5 hours later. 21 hours post transfection cells were fixed and stained with the indicated antibodies for immunocytochemistry (B) Quantification of endogenous TAF6δ expressing cells transfected with splice site-switching antisense oligonucleotides. Results are expressed as the percentage of cells displaying a clear TAF6δ punctate staining on a total of at least 500 cells. (C) Translation of exogenous TAF6δ is induced by modified antisense RNA oligonucleotides. Schematic representation of plasmid pASTAF6 containing sequences derived from the TAF6 cDNA (white) or from genomic DNA (grey). HeLa cells were first transfected with pASTAF6 and 19 hours later with splice site switching oligonucleotides and treated with MG132 and ZVAD-FMK 6 hours after this second transfection. 38 hours post-transfection protein extracts from cells were analyzed by immunoblotting with monoclonal antibodies directed against TFIID subunits.

We next investigated the influence of the splice site switching oligonucleotides on the levels of TAF6δ and TAF6α proteins. TAF6 was detected by immunocytochemistry using monoclonal antibodies that recognize an epitope present in all of the known isoforms of TAF6. HeLa cells treated with negative control oligonucleotides showed strong TAF6 staining throughout the entire nucleoplasm (Fig. 2A). The nuclear total TAF6 immunofluorescent signal is diminished in cells treated with SSOs that increase TAF6δ mRNA production (Fig. 2A), presumably due to decreased expression of TAF6α (see also below). TAF6δ was detected by immunofluorescence with monoclonal antibodies that specifically recognize the delta TAF6 isoform [39]. HeLa cells transfected with negative control antisense oligonucleotides exhibited undetectable cellular staining with anti-TAF6δ monoclonal antibodies (Fig. 2A). In contrast, transfection of HeLa cells with oligonucleotides that induce TAF6δ mRNA expression resulted in punctate nuclear staining (Fig. 2A). We further quantified the influence of antisense treatment by scoring the number of cells displaying clear nuclear TAF6δ immunofluorescent signals. We found that treatment with the Taf6 AS1 oligonucleotide resulted in nearly ∼10 fold more cells with TAF6δ staining compared to control treated cells (Fig. 2B). As a further control of specificity, oligonucleotide Bcl-x AS was transfected and caused no increase in nuclear TAF6δ immunofluorescent staining (Fig. 2B). We conclude that TAF6δ protein in discrete nuclear loci is significantly increased by SSO targeting of the TAF6 pre-mRNA.

Immunofluorescence experiments measure the levels of detectable antigen in fixed cells. Since the levels of total cellular protein can potentially differ from the antigenically recognizable levels of protein we employed immunoblotting under denaturing conditions to directly examine the effect of TAF6δ-inducing oligonucleotides on the translation of the TAF6δ mRNA in treated cells. Endogenous TAF6δ is undetectable by Western blotting of extracts from HeLa cell due to its rapid turnover by the proteasome (data not shown) and by caspase-dependent cleavage in apoptotic cells [39]. We therefore developed a *TAF6* minigene plasmid that is responsive to SSO (pASTAF6, Fig. 2C). Immunoblots on total protein extracts from HeLa cells transfected with the spliceable minigene construct and later treated with TAF6δ-inducing oligonucleotides resulted in a marked increase in TAF6δ protein levels, with a corresponding reduction in TAF6α (Fig. 2C). The levels of two other TFIID subunits, TAF5 and TBP, remained relatively constant. These data demonstrate a selective induction of TAF6δ translation and concomitant reduction in TAF6α levels by TAF6δ-inducing SSOs.

### Endogenous TAF6δ expression causes apoptosis in HeLa cells

**Figure 3:**
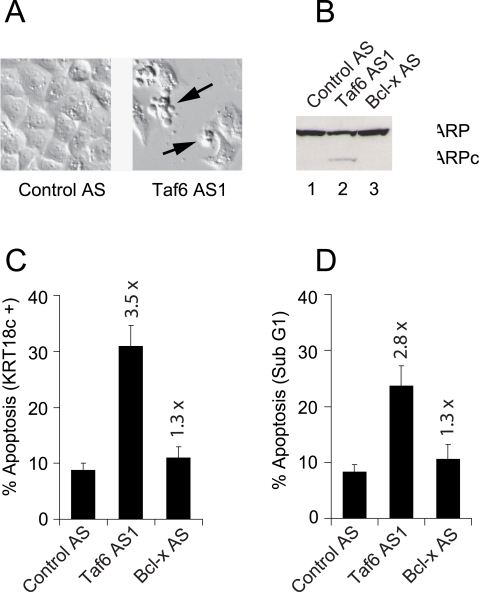
Expression of endogenous TAF6δ causes cell death by apoptosis. HeLa cells were transfected with antisense oligonucleotides that induce endogenous TAF6δ (Taf6 AS1), scrambled control oligonucleotides (Control AS), or oligonucleotides that induce endogenous Bcl-xS expression (Bcl-x AS). (A) 24 hours post transfection cells were observed by differential interference contrast microscopy. (B) Proteins were extracted from transfected cells and subjected to immunoblot analysis with anti-PARP monoclonal antibodies. PARPc indicates caspase cleaved PARP. (C) The percentage of apoptotic cells was analyzed by flow cytometry using monoclonal antibodies that detect caspase cleaved cytokeratin-18. (D) The percentage of apoptotic cells was analyzed by flow cytometry to detect sub G1 DNA content. The values indicated above the data bars are the fold induction of apoptosis with respect to Control oligonucleotide-treated cells.

We next investigated the physiological consequences of SSO-induced endogenous TAF6δ expression in HeLa cells. The transfection of HeLa cells with a scrambled antisense oligonucleotide resulted in no obvious morphological changes or changes in cell number when visualized by light microscopy (Fig. 3A, left image). In stark contrast, TAF6δ-inducing SSO resulted in an obvious loss of adherent cells and produced significant numbers of cells that exhibit the classical features of apoptosis, including membrane blebbing (Fig. 3A, right image). To obtain further evidence that TAF6δ induction causes apoptosis we measured cleavage of the well-known caspase substrate PARP-1, since activation of the caspase protease cascade is a defining biochemical feature of apoptosis. Immunoblotting revealed readily detectable cleavage of PARP-1 in cells when TAF6δ was induced (Fig. 3B, lane 2). As an additional control for the specificity of the Taf6 AS1 oligonucleotide, we used a Bcl-xS-inducing SSO. Consistent with a previous report [47], the induction of Bcl-xS expression has little effect on apoptosis in HeLa cells (Fig. 3B, lane 3). To further substantiate and quantify TAF6δ-induced apoptosis we employed flow cytometry to measure the levels of caspase-cleaved cytokeratin-18 (KRT18c), another established marker of apoptosis [48]. Treatment of HeLa cells with the Taf6 AS1 oligonucleotide resulted in a 3.5 fold increase in KRT18c positive cells (Fig. 3C). As an independent quantification of apoptosis, we employed flow cytometry to measure the level of Sub-G1 DNA content. This assay showed that TAF6δ induction resulted in a 2.8 fold increase in apoptosis whereas Bcl-xS induction resulted in a 1.3 fold increase in apoptosis in HeLa cells (Fig. 3D). Thus, four distinct assays show that the induction of endogenous TAF6δ triggers a robust apoptotic response in HeLa cells.

### TAF6δ induces apoptosis in the absence of p53

**Figure 4:**
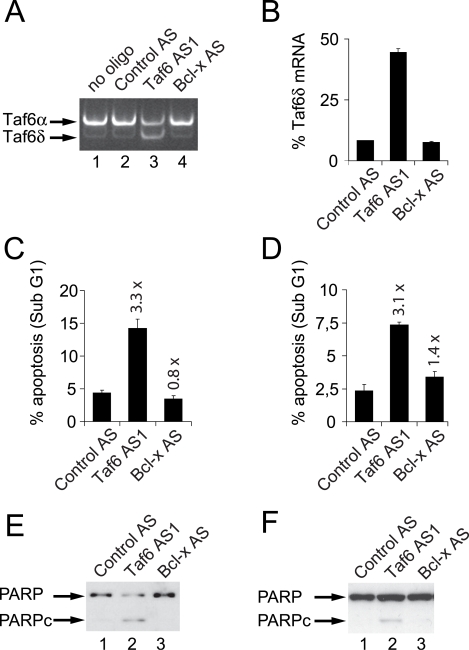
TAF6δ induces apoptosis in cancer cell lines lacking p53. Saos-2 human bone osteosarcoma cells, that do not express p53, were transfected with antisense oligonucleotides that induce endogenous TAF6δ (Taf6 AS1), scrambled control oligonucleotides (Control AS), or oligonucleotides that induce endogenous Bcl-xS expression (Bcl-x AS). (A) 24 hours post transfection total RNA was isolated for analysis by RT-PCR with primers that amplify both the TAF6α and TAF6δ mRNAs. (B) RT-PCR products were analyzed on an Agilent Bioanalyzer. Error bars indicate the standard deviation of three independent transfections. (C) Percentage of apoptotic Saos-2 cells was analyzed by flow cytometry to detect sub G1 DNA content. Error bars indicate the standard deviation of three independent transfections. (D) As in C except that A549 human lung carcinoma cells, that express wild-type p53, were transfected. (E) As in C except that proteins were extracted from transfected Saos-2 cells and subjected to immunoblot analysis with anti-PARP monoclonal antibodies. PARPc indicates caspase cleaved PARP. (F) As in E except with A549 cells.

**Figure 5:**
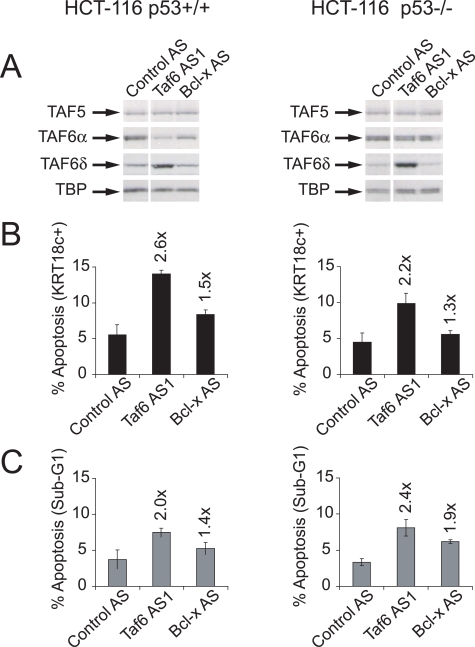
p53 is dispensable for TAF6δ-induced apoptosis. (A) Western blot analysis of TFIID subunits following TAF6 and Bcl-x SSO in HCT-116 p53 +/+ (left panels) and HCT-116 p53 −/− cells (right panels). HCT-116 cells were transfected first with plasmid pASTAF6 (see Fig. 2C) and 19 hours later with antisense oligonucleotides that induce endogenous TAF6δ (Taf6 AS1), scrambled control oligonucleotides (Control AS), or oligonucleotides that induce endogenous Bcl-xS expression (Bcl-x AS). Total cells extracts were prepared and separated on 10% SDS-PAGE, followed by immunoblotting with anti-TFIID subunit antibodies as indicated with arrows. (B) The percentage of apoptotic cells was analyzed by flow cytometry using monoclonal antibodies that detect caspase cleaved cytokeratin-18. (C) The percentage of apoptotic cells was analyzed by flow cytometry to detect sub G1 DNA content. The values indicated above the data bars are the fold induction of apoptosis with respect to Control oligonucleotide-treated cells. Error bars indicate the standard deviation of three independent transfections.

The tumor suppressor p53 interacts physically and functionally with TAF6α (see Introduction). Mutations in the p53 pathway are thought to allow human tumor cells to escape apoptotic death and therefore allow cancer development [6]. It was therefore of fundamental importance to establish whether TAF6δ-induced apoptosis can occur in the absence of p53. To address whether TAF6δ-dependent apoptosis requires p53 we transfected the Saos-2 osteosarcoma cell line that is devoid of a functional *p53* gene [49] with oligonucleotide Taf6 AS1. Transfection of Taf6 AS1, but not Bcl-x AS into Saos-2 cells effectively increased endogenous TAF6δ mRNA levels (Fig. 4A, lane 3). Analysis of the RT-PCR results showed an approximately ∼5 fold induction in the TAF6δ/TAF6α+δ mRNA ratio (Fig. 4B). The expression of TAF6δ induced a 3.3 fold increase in apoptosis in Saos-2 as measured by Sub-G1 DNA content (Fig. 4C). Similar results were obtained in another cell line (H1299 lung carcinoma) that does not contain p53 (data not shown). Because HeLa cells have impaired p53 function due to the expression of the Human Papilloma Virus E6 gene product [50], we also compared the efficiency of induction of apoptosis in the A549 lung carcinoma cells because they express wild type p53. Taf6 AS1 transfection increased apoptosis by 3.1 fold (Fig. 4D), whereas Bcl-x AS transfection caused no increase in apoptosis over background levels (Fig. 4D). The fact that Saos-2 are at least as susceptible as A549 cells to TAF6δ-induced programmed cell death was further verified by measuring PARP-1 cleavage by immunoblotting (Fig. 4E versus 4F). To reinforce the fact that p53 is dispensable for TAF6δ-induced apoptosis, we employed the HCT-116 human colon carcinoma cell line and its isogenic counterpart HCT-116 p53 −/− in which the p53 gene has been deleted by homologous recombination [51]. The induction of apoptosis by TAF6δ in isogenic cells lacking p53 is equally robust as in wild-type cells, as judged by significant increases in both caspase-cleavage of cytokeratin-18 (Fig. 5B) and Sub-G1 DNA content (Fig. 5C). The induction of TAF6δ protein levels by the SSO strategy was efficient in both cells lines (Fig. 5A). The results demonstrate that p53 is dispensable for TAF6δ-induced cell death. We conclude that TAF6δ controls apoptosis irrespective of cellular p53 status.

### TAF6δ activates gene expression independently of p53

TAF6δ can induce apoptosis of several cancer cell lines independent of their p53 status. We have previously shown that TAF6δ can bind the TFIID subunits TAF1, TAF5, TBP and TAF12 *in vitro*, and forms a TFIID-like complex that contains several TAFs but lacks TAF9 (TFIIDπ) *in vivo* [39]. *TAF6* is an essential gene that plays a broad role in the regulation of transcription programs (see Introduction). To investigate whether TAF6δ can regulate transcription, with an emphasis on potentially p53-independent transcription, we employed genome-wide microarray technology. The transcriptional effects of TAF6δ are technically difficult to measure because endogenous TAF6δ is not induced in all cells during antisense transfection (Fig. 2B) and because endogenous TAF6δ-expressing cells are lost rapidly from the culture by apoptosis (Fig. 3). In order to achieve maximal sensitivity we chose a recently developed microarray technology based on chemiluminescent detection and longer oligonucleotide probes (60 nucleotides), that has been shown to provide increased signal dynamic range and higher sensitivity when compared to traditional microarray technologies [52], [53]. The microarrays used represent 27,868 annotated human genes (Material and Methods).

The design of the microarray experiments enables detection of direct TAF6δ target genes without excluding potentially informative rapid secondary changes in mRNA levels. Wild-type HCT-116 and their p53-null isogenic counterparts (HCT-116 p53 −/−) were transfected with oligonucleotides Taf6 AS2 and Control AS, and total RNA was isolated and subjected to microarray analysis after 24 hours. The scrambled control oligonucleotide was employed as a reference to exclude any non-specific changes in gene expression due to the transfection protocol or the introduction of exogenous oligonucleotide into cells. Biological triplicates (three independent transfections) were performed for each condition and statistical analysis and filtering was performed, as detailed in Materials and Methods, to identify significantly (P<0.05) regulated mRNAs.

**Figure 6:**
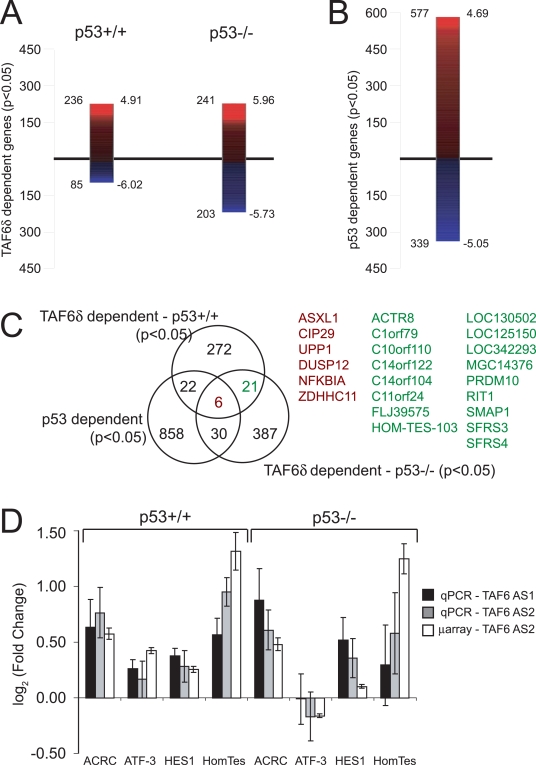
Endogenous TAF6δ expression induces apoptosis in the absence of p53. (A) Transcriptome analysis following TAF6 SSO in HCT-116 p53+/+ and p53−/− cells. Expression levels of mRNAs from cells treated with oligonucleotide Taf6AS2 were individually compared to control oligonucleotide-treated HCT-116 samples by genome-wide microarray analysis. The absolute number of probes detecting statistically significant (p<0.05) up- or down-regulation following splice-site selection is shown to the left of each bar; the maximum positive or negative logarithmic (base two) fold-change is shown to the right. The red gradient indicates positive, and the blue gradient negative fold changes in expression. (B) Transcriptome analysis following TAF6 SSO in HCT-116 cells and selecting for those genes that show significant regulation between the HCT-116 p53 +/+ versus HCT-116 p53 −/− cells irrespective of TAF6δ expression. Labels as in (A). (C) Venn-Diagram displaying the overlap between the three different target gene repertoires shown in (A) and (B). (D) Verification of gene expression changes by quantitative real-time RT-PCR. HCT-116 p53+/+ and HCT-116 p53−/− cells were transfected with two distinct antisense oligonucleotides that induce endogenous TAF6δ; Taf6 AS1 (grey bars), Taf6 AS2 (black bars). White bars indicate values from microarray experiments for comparison. 18 hours post-transfection total RNA was extracted and the levels of the indicated mRNAs were analyzed by quantitative real-time PCR with respect to the levels from cells treated with the control oligonucleotide. Error bars indicate standard deviation of three independent transfections.

The induction of endogenous TAF6δ in wild-type HCT-116 cells resulted in significant changes in the levels of 321 mRNAs out of a total of 27,868 independent genes measured by microarray analysis (Fig. 6A). The induction of endogenous TAF6δ in HCT-116 lacking p53 expression resulted in significant changes in the levels of 444 mRNAs. In both cells the majority of mRNAs are increased in response to TAF6δ. These data establish that TAF6δ acts primarily as a positive regulator of gene expression and rule out the possibility that TAF6δ-induced cell death is a result of a global reduction in mRNA transcription.

TAF6α physically interacts with p53 [34], yet TAF6δ induces apoptosis in cells lacking p53. We therefore analyzed the microarray data to determine whether TAF6δ can control gene expression independently of p53. The p53-dependent genes were identified by filtering for genes that are significantly changed in the wild-type HCT-116 versus HCT-116 p53 −/− in both the presence of the TAF6 SSO or a scrambled control oligonucleotide (Fig. 6B). As expected, well-established p53 target genes, including FAS [54], FDXR [8], SESN1 [55] and p21/CDKN1A [56] were found in the p53-dependent gene set (Supplementary Data File S3), confirming the sensitivity and accuracy of the microarray methodology. We focused on the identification of genes regulated in both wild-type HCT-116 and HCT-116 p53 −/− because these mRNAs represent candidates for genes that function to induce p53-independent apoptosis. The different gene sets significantly regulated by TAF6δ in wild-type and p53 negative HCT-116 cells, as well as the p53-dependent genes, are shown by Venn diagrams in Fig. 6C. The absolute numbers of TAF6δ-dependent genes is underestimated when compared to p53-dependent genes because the two gene sets are derived from very technically different approaches. p53-dependence is defined here through the use of an isogenic cell line in which p53 expression is eliminated completely in 100% of cells by genetic ablation through homologous recombination. In contrast TAF6δ-dependency is defined by the induction of endogenous TAF6δ via transient transfection with splice switching oligonucleotides, that occurs only partially (Fig. 1C) and in fraction of the cells (Fig. 2B). Nonetheless, the analysis revealed 21 TAF6δ-dependent, p53-independent genes (Fig. 6C). To independently validate the TAF6δ-dependent genes we selected 4 genes (and the internal control FNBP4) for real-time quantitative RT-PCR analysis. One gene (HOM-TES-103) is within our P-value cut-of (P<0.05), another (ACRC) is slightly outside the P-value cut-off (P = 0.0698), and a third (HES1) substantially outside our cutoff (P = 0.2012). ACRC, HES1 and HOM-TES-103 were induced by TAF6δ in wild-type p53 HCT-116 cells as well as HCT-116 p53 −/− cells (Fig. 6D). We also verified the expression of ATF3, since it represents the distinct class of genes that are regulated by TAF6δ only in the presence of p53. ATF3 induction was documented in HCT-116 cells expressing p53 but not in p53-null HCT-116 cells, as validated by real-time RT-PCR (Fig. 6D). To reinforce the specificity of all of these effects, we employed two distinct TAF6δ-inducing SSOs, both of which caused comparable changes in expression of the four genes tested (Fig. 6D). These results confirm that TAF6δ can induce gene expression independently of the tumor suppressor p53.

## Discussion

Here we have combined splice-switching oligonucleotides (SSOs) with high-sensitivity genome-wide microarrays to shed light on the function of endogenous TAF6δ. By experimentally inducing endogenous TAF6δ expression we show that TAF6δ triggers robust cell death in several cancer cell types. The expression of the p53 tumor suppressor is dispensable for TAF6δ-dependent cell death and gene expression in several cell lines. The data establish the TAF6δ pathway as an important signaling hub that can control apoptosis in the absence of p53. Our microarray results show that TAF6δ expression activates genes, such as HOM-TES-103, HES1 and ACRC independently of p53. The 21 genes we identify here that are controlled by TAF6δ independently of p53 represent candidate genes that could mediate TAF6δ-dependent apoptosis. The majority of genes identified through the unbiased microarray approach are of as yet unknown biological function; a finding that is not unexpected given that TAF6δ represents a newly discovered signaling pathway. Therefore, further work will be required to determine their contributions to the apoptotic program. Given the fact that TAF6δ activates minimally hundreds of genes, it is improbable that any single gene product could account fully for TAF6δ-driven apoptosis. The microarray data presented here represent, to our knowledge, the first documentation of changes in gene expression due to induction of an endogenous TFIID subunit. Based on the increases in gene expression we have demonstrated here, we propose a model in which TAF6δ drives a pro-apoptotic transcription program to initiate the apoptotic cascade.

Despite intensive efforts, the molecular mechanisms by which the TAFs specifically influence gene expression remain obscure. The advantage of the SSO strategy employed here is that it reveals for the first time a pro-apoptotic role for endogenous TAF6δ levels. As is the case for all physiological alternative splicing events, the TAF6δ isoform is produced with a concomitant reduction in the levels of the major alpha isoform. The reduction in TAF6α may contribute to changes in gene expression during the SSO-enforced as well as the normal physiological switch from alpha to delta expression. It is, however, important to note that the overexpression of TAF6δ alone is sufficient to induce apoptosis in cells that express endogenous TAF6α [39]. Therefore, although decreased TAF6α expression necessarily accompanies increased TAF6δ expression, all the available evidence indicates that the increase of the minor TAF6δ isoform is a critical molecular event triggering apoptosis.

Mounting evidence suggests that functionally distinct forms of TFIID exist [57], and that TFIID composition is dynamic [58]. Recently, the dynamic nature of core promoter recognition complexes *in vivo* has been underscored by the observation of a drastic replacement of the cellular pools of canonical TFIID with a complex containing TRF3/TAF3 during myogenesis [59]. The number of combinatorial possibilities for more subtle changes within the TFIID complex itself continues to expand with the discovery of new TAF isoforms; TAF4 and TAF4b possess distinct target gene specificities [60], TAF9 and TAF9b can both incorporate into TFIID but are functionally non-redundant [61], and TAF1-2 and TAF1-4 are signal-inducible and functionally distinct splice variants of TAF1 [62], [63]. To date, however, the functional consequences for changes in the cellular levels of endogenous TFIID subunits have remained unknown. The current findings show that the induction of a single TFIID subunit within living cells can orchestrate gene expression programs to alter cell physiology. TAF6δ mRNA levels can be dramatically induced in HL-60 cells after differentiation by retinoic-acid, demonstrating at least one physiological situation where TAF6δ is induced in living cells [39]. Recent genetic evidence that TAF12 is required for ethylene-responsive transcription in plants [64] further argues that TFIID is a signal-responsive transcription factor. Therefore, in addition to its known functions in core promoter recognition and co-activation, TFIID represents a platform that integrates cellular signals with the control of gene expression.

The pro-apoptotic transcription factor p53 plays a central role in genome surveillance and tumor suppression. The p53 protein is not required for cell viability and indeed is lost or mutated in roughly 50% of tumors [6]. Even in animal models where functional p53 can be restored by gene therapy, tumors readily attain resistance to p53 due to inactivation of p19*^ARF^* or p53 itself [65]. The efficient induction of cell death in several different tumor cell lines by SSO targeting of *TAF6*, independent of their p53 status, provides a proof-of-principle that the TAF6δ pathway can be exploited to kill tumor cells. The data presented here define the TAF6δ signaling hub as able to control apoptosis without p53, but with interconnections to the p53 pathway including several shared target genes, as revealed by transcriptome-wide microarray analysis (Fig. 6B). Unlike p53, TAF6 is essential for viability in all organisms studied [26], [27], [28], [29]. Furthermore, targeting *TAF6* results in a substantially more robust apoptotic response than targeting another apoptotic gene, *Bcl-x* in several tumor cell lines (Fig.s 3, 4 & 5). Further characterization of the TAF6δ signaling hub may therefore provide novel therapeutic avenues to induce controlled tumor cell death irrespective of their p53 status.

The TAF6δ pathway remains an orphan pathway since the precise molecular trigger that induces TAF6δ expression in the physiological context is currently unknown. The fact that TAF6δ can act downstream of p53 to control gene expression, and that TAF6δ can dictate cell death versus life decisions of human cells, evoke the possibility that this newly defined pathway could be subject to deregulation in certain cancer cells. In this light, it is intriguing that expression levels of TAF6 have been correlated with the inflammatory breast cancer phenotype [66], and isoform specific enrichment of a TAF6 splicing variant has been reported in breast cancer [45]. Experiments to identify the upstream signals that control TAF6δ expression *in vivo* in healthy tissues, as well as to uncover the potential role of mutations to the TAF6δ pathway in cancer are ongoing in our laboratory.

## Supporting Information

Figure S1 (A) Time course for antisense RNA-mediated TAF6δ mRNA induction. Transfections and RT-PCR were performed as in Fig. 1C except that RNA was extracted at various times (x-axis) after transfection. (B) Dose-dependent antisense mediated induction of TAF6δ mRNA expression. As in B, with different concentrations of oligonucleotides transfected indicated on the x-axis.
(0.71 MB EPS)

Supplementary Data File S1 Genes changing in response TAF6delta in HCT-116 p53 +/+ cells
(0.05 MB TXT)

Supplementary Data File S2 Genes changing in response to TAF6delta in HCT-116 p53 −/−
(0.07 MB TXT)

Supplementary Data File S3 Genes differentially expressed in the HCT-116 p53 +/+ cells versus in HCT-116 p53 −/− cells both in cells treated with control oligonucleotide and TAF6delta -inducing oligonucleotides
(0.24 MB TXT)

Supplementary Data File S4 Genes differentially regulated by TAF6delta in both HCT-116 p53 +/+ cells and HCT-116 p53 −/− cells
(0.01 MB TXT)
